# Development of a Gradient HPLC Method for the Simultaneous Determination of Sotalol and Sorbate in Oral Liquid Preparations Using Solid Core Stationary Phase

**DOI:** 10.1155/2015/806736

**Published:** 2015-03-24

**Authors:** Ludmila Matysova, Oxana Zahalkova, Sylva Klovrzova, Zdenka Sklubalova, Petr Solich, Lukas Zahalka

**Affiliations:** ^1^Department of Analytical Chemistry, Faculty of Pharmacy in Hradec Kralove, Charles University in Prague, 500 05 Hradec Kralove, Czech Republic; ^2^Department of Pharmaceutical Technology, Faculty of Pharmacy in Hradec Kralove, Charles University in Prague, 500 05 Hradec Kralove, Czech Republic; ^3^Hospital Pharmacy, University Hospital in Motol, 150 06 Prague 5, Czech Republic

## Abstract

A selective and sensitive gradient HPLC-UV method for quantification of sotalol hydrochloride and potassium sorbate in five types of oral liquid preparations was developed and fully validated. The separation of an active substance sotalol hydrochloride, potassium sorbate (antimicrobial agent), and other substances (for taste and smell correction, etc.) was performed using an Ascentis Express C18 (100 × 4.6 mm, particles 2.7 *μ*m) solid core HPLC column. Linear gradient elution mode with a flow rate of 1.3 mL min^−1^ was used, and the injection volume was 5 *µ*L. The UV/Vis absorbance detector was set to a wavelength of 237 nm, and the column oven was conditioned at 25°C. A sodium dihydrogen phosphate dihydrate solution (pH 2.5; 17.7 mM) was used as the mobile phase buffer. The total analysis time was 4.5 min (+2.5 min for reequilibration). The method was successfully employed in a stability evaluation of the developed formulations, which are now already being used in the therapy of arrhythmias in pediatric patients; the method is also suitable for general quality control, that is, not only just for extemporaneous preparations containing the mentioned substances.

## 1. Introduction

Sotalol (SOT) is a Class III antiarrhythmic agent that prolongs the QT interval and exhibits beta-adrenergic blocking properties. SOT has been widely used in the management of atrial arrhythmias for several decades including patients in the pediatric age group and those with congenital heart disease. In pediatric patients, SOT has proven efficacy in suppressing supraventricular arrhythmias and maintaining a sinus rhythm with recurrence-free intervals of >80% and has also been used in the management of ventricular arrhythmias with more modest efficacy [1].

Potassium sorbate (SORB) is an antimicrobial preservative with antibacterial and antifungal properties and is used in pharmaceuticals, foods, enteral preparations, and cosmetics. In general, SORB is used at concentrations of 0.1–0.2% in oral and topical formulations. Potassium sorbate is used in approximately twice as many pharmaceutical formulations as sorbic acid due to its greater solubility and stability in water. As with sorbic acid, potassium sorbate exhibits minimal antibacterial properties in formulations with pH values higher than 6 [2].

There are no registered medicinal products containing sotalol suitable for administration in pediatric patients and available in the European Union (EU) member states and selected non-EU countries (Supplement) (see Supplementary Material available online at http://dx.doi.org/10.1155/2015/806736) [3]. Pediatric formulations have many specific characteristics. The most important one is the ability to administer dosages of an active substance in variable and precise amounts according to the actual weight of a child. In addition, the dosage form has to be easily swallowed [4, 5]. When no appropriate dosage form is commercially available, the most suitable alternative is the use of oral liquid extemporaneous preparations with an antimicrobial agent for older children and with no antimicrobial agent for infants. The cooperation between the Department of Pharmaceutical Technology (Charles University, Faculty of Pharmacy, Hradec Kralove) and the Hospital Pharmacy (University Hospital in Motol, Prague) has led to the development of five versions of oral liquid preparations with sotalol hydrochloride as the active substance and potassium sorbate as the preservative. The HPLC determination of sotalol has been previously reported [6–10], but the simultaneous determination of sotalol and sorbate in various matrices (e.g., sirupus simplex~sucrose syrup, saccharine, and citric acid) in a liquid dosage has not been previously published. The aim of this study was to develop and validate a selective and rapid method using standard HPLC system for the determination of sotalol hydrochloride (i.e., the active substance) and potassium sorbate (i.e., the antimicrobial agent) and their separation from other present substances in newly developed pediatric oral preparations and its application for stability study. In order to achieve total separation of sotalol, sorbate, and other analytes that possess different chromatographic properties at the lowest possible analysis time at standard HPLC system, modern solid core columns and gradient elution were adopted during method development.

Columns of solid core particles exhibit unusual chromatographic efficiency. Presumably, this is due to the ability to form very homogeneous packed beds as a result of an extremely narrow particle size distribution and higher particle density. Solid core particles exhibit highly improved mass transfer (kinetic) effects because of the thin porous shell surrounding a solid core, allowing solutes to rapidly diffuse in and out of the porous structure containing the stationary phase for interaction. Columns of the solid core particles (2.7 *μ*m) exhibit theoretical plates nearly comparable to those of sub-2-micron totally porous particles, but with much reduced pressure requirements and thus it is possible to use them at standard HPLC systems [11].

## 2. Materials and Methods

### 2.1. Materials and Chemicals

Sotalol hydrochloride (Fagron, Olomouc, Czech Republic) and potassium sorbate (Dr. Kulich Pharma, Hradec Kralove, Czech Republic) were used as the standards. Ethylparaben (Sigma-Aldrich, Steinheim, Germany) was used as an internal standard (IS). Sodium dihydrogen phosphate dihydrate (Sigma-Aldrich, Steinheim, Germany), orthophosphoric acid (Merck, Darmstadt, Germany), acetonitrile (ACN) gradient grade (Sigma-Aldrich, Steinheim, Germany), methanol (MeOH) gradient grade (Sigma-Aldrich, Steinheim, Germany), and tetrahydrofuran (THF) Chromasolv (Sigma-Aldrich, Steinheim, Germany) were used to prepare the mobile phase. Water for the sample and mobile phase preparation was purified by Milli-Q Integral 15 system with 0.22 *μ*m output filter. Nylon membrane filters (0.20 *μ*m) were used for mobile phase filtration (Albet, Dassel, Germany). Nylon membrane filters (0.22 *μ*m) (Vitrum, Prague, Czech Republic) and 2 mL syringes (Chirana T. Injecta, Stara Tura, Slovak Republic) were used to filter the samples. A 1000 *μ*L Transferpette micropipette (Brand, Wertheim, Germany) was used. Formulations F1–F5 (and respective blank solutions), which contain sotalol hydrochloride (5 mg mL^−1^), potassium sorbate (1 mg mL^−1^), and excipients (e.g., water for injection, sirupus simplex, citric acid, disodium hydrogen phosphate dodecahydrate, and sodium saccharine), were obtained as extemporaneous preparations from the Hospital Pharmacy at the University Hospital in Motol, Prague, Czech Republic.

### 2.2. Instrumentation and Chromatographic Conditions

The chromatographic analysis was performed on an integral system Shimadzu LC-2010C (Shimadzu, Kyoto, Japan). The following chromatographic columns were tested during method development: Ascentis Express C18 (150 × 4.6 mm, particles 2.7 *μ*m), Ascentis Express C18 (100 × 4.6 mm, particles 2.7 *μ*m), and Ascentis Express Phenyl-Hexyl (100 × 4.6 mm, particles 5 *μ*m). An Ascentis Express C18 (100 × 4.6 mm, particles 2.7 *μ*m) column was finally chosen for the method validation and stability testing. The dual absorbance UV/Vis detector was set to a wavelength of 237 nm. Linear gradient elution (Table 1) with a flow rate of 1.3 mL min^−1^ was used. A column oven was conditioned at 25°C. The injection volume was 5 *μ*L and analysis time was 4.5 minutes (7 minutes with reequilibration time incl.).

### 2.3. Preparation of Buffer Component of Mobile Phase (Approximately 1 L)

2.76 g of sodium dihydrogen phosphate dihydrate was dissolved in 1 L of ultrapure water. An orthophosphoric acid solution (6%) was used to adjust the pH to 2.5 (±0.05). The mobile phase buffer was filtered through a nylon membrane filter (0.20 *μ*m) using a Millipore glass filter holder. The mobile phase buffer was used immediately after preparation or stored in the refrigerator in closed borosilicate glass bottles for a maximum of 24 hours.

### 2.4. Preparation of Stock, IS, Standard, Sample, and Blank Solutions

The preparation of the stock, IS, standard, sample, and blank solutions is described in Table 2.

## 3. Results and Discussion

### 3.1. Method Development

**Figure 1:**
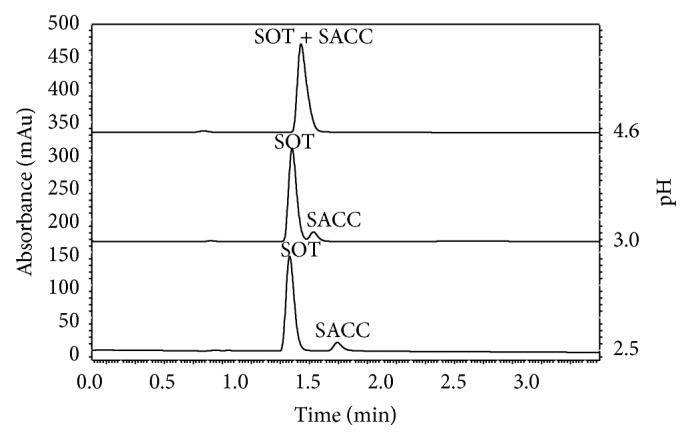
Significance of mobile phase buffer pH controlling in SOT/SACC separation; pharmaceutical formulation F4 (1.000 mL of pharmaceutical preparation diluted to 25.00 mL); injection volume 5 *μ*L; mobile phase flow 1.3 mL min^−1^; linear gradient (ACN: 10% to 60% in 4 minutes); UV/Vis detector wavelength 237 nm; column oven 25°C.

The initial chromatographic conditions and mobile phase composition were chosen to be similar to those used by Delamoye et al. for the separation of thirteen *β*-blockers [12]. C18 stationary phase column with solid core 2.7 *μ*m particles, 4.6 mm i.d., and 100 mm length was initially tested. A mobile phase consisting of sodium dihydrogen phosphate dihydrate (pH 3.8; 17.7 mM)-ACN (65 : 35, v/v) did not provide separation of the sotalol peak from the dead volume peak. An increase in the phosphate buffer (pH 3.8; 17.7 mM) component led to a desirable increase in the sotalol retention. Phosphate buffer (pH 3.8; 17.7 mM)-ACN (80 : 20 and 85 : 15, v/v) only provided partial separation of sotalol from the dead volume peak. Phosphate buffer (pH 3.8; 17.7 mM)-ACN (90 : 10, v/v) was sufficient for proper sotalol retention. The addition of THF was tested to observe possible positive effects on peak shape. Unfortunately, THF addition did not provide any advantages and led to a rapid increase in the baseline noise and drift. The use of MeOH instead of acetonitrile also led to a less stable baseline as well as an undesirable increase in the system back pressure. Avoiding the phosphate buffer and using only ACN-water mobile phases caused unacceptable peak fronting and tailing. Therefore, the buffer is necessary for maintaining good peak shapes and separation. Isocratic elution with the phosphate buffer (pH 3.8; 17.7 mM)-ACN (90 : 10, v/v) mobile phase cannot be used due to a significant increase in the analysis time caused by different retention properties of sotalol (base) and sorbate (acid). Under acidic conditions sotalol is in ionized form and thus it is not well retained on the stationary phase; opposite sorbate is in nonionized form and it is therefore significantly retained on the column. Different gradient curve profiles were tested, and a linear gradient was chosen because it resulted in the lowest baseline drift. A terminal gradient concentration of ACN was tested up to 70%, but a maximum usable concentration of 60% was required to maintain a straight baseline. Gradient elution with initial phosphate buffer (pH 3.8; 17.7 mM)-ACN (from 90 : 10 to 40 : 60, v/v) could be used for the separation of sotalol and sorbate. Unfortunately, these conditions cannot be used for analysis of preparations containing the artificial sweetener saccharine (SACC) due to its coelution with the sotalol peak. An increase in the temperature up to 60°C or the use of an Ascentis Express Phenyl-Hexyl column did not provide any favorable changes in the selectivity and using of Ascentis Express C18 (150 × 4.6 mm, 2.7 *μ*m particles) also did not provide sufficient SOT–SACC separation. An elevated temperature resulted even in a decreased resolution of the SOT and SACC peaks. Several buffer pH values (4.6; 3.8; 3.0; 2.5; and 2.0) were tested (Figure 1). Using 4.6 or 3.8 pH buffers caused coelution of SOT/SACC, pH 3.0 buffer provided reasonable separation of the SOT/SACC peaks (resolution = 1.44), and finally the 2.5 buffer led to complete separation of the mentioned analytes to the baseline (resolution > 1.5). The pH 2.0 buffer also provided total SOT/SACC separation but it is not recommended due to an expected decrease in the column lifetime. These experimental results correspond to the theoretical useful pH range of phosphate buffer which is 2.1–3.1 [13]. Methylparaben, ethylparaben, propylparaben, butylparaben, paracetamol, and salicylic acid were tested as possible internal standards (IS). Paracetamol was coeluted with the dead volume peak, methylparaben and salicylic acid were not sufficiently separated from the sorbate peak, and propylparaben with butylparaben was eluted with unfavorable long retention times. Ethylparaben was finally chosen as the IS because it is stable in solution, inexpensive, and well separated from all of the analytes in the oral preparations. In addition, ethylparaben exhibits good UV absorption in UV. Various concentrations of the sample solution and injection volumes were tested to ensure a suitable tailing factor and sufficient response (absorbance). The UV spectra of sotalol and sorbate were obtained with a UV/Vis DAD spectrophotometer, and the tested wavelengths of the UV/Vis absorbance HPLC detector ranged from 200 to 300 nm. Finally, the wavelength was set to 237 nm to ensure good sensitivity, as well as low baseline noise.

### 3.2. Sample Preparation Development

The simple method known as “dilute and shoot” was used for sample preparation. The pharmaceutical preparation was diluted 25 times (i.e., 1.000 mL of the preparation with 1.000 mL of the IS stock solution was diluted to 25.00 mL with a mixture of ACN-water (30 : 70, v/v)) to avoid previously reported matrix effects of the liquid pharmaceutical formulations [14]. The standard solution was prepared in the same way as the sample solution using a stock solution of the standards. The concentration of SOT, SORB, and EP was selected to ensure the same concentration level in the sample and standard solutions. An increase in the ACN component (e.g., to 50%) led to rapid deterioration of the peak shapes, especially significant fronting of the SOT and SACC peaks. Therefore, an ACN concentration higher than 30% is not practical.

### 3.3. Method Validation

The method was validated according to ICH Q2 (R1) guidelines [15]. The system suitability (i.e., repeatability of retention times and areas, number of theoretical plates, resolution, and tailing factor), precision, linearity, accuracy, selectivity, and robustness were evaluated during method validation (Table 3). The parameters accuracy, precision, and selectivity were performed and evaluated for all five pharmaceutical formulations.

#### 3.3.1. System Suitability Test (SST)

SST was performed on a standard solution that was injected into the column six times. The reported values are arithmetic means of six injections.

#### 3.3.2. Precision

Six sample solutions were prepared from each of the five preparations. Each sample was injected three times. The final results are reported as relative standard deviations (R.S.D.) of the SOT/EP and SORB/EP ratios of the peak areas.

#### 3.3.3. Linearity

A calibration curve was created using 6 points that covered the concentration range of sotalol hydrochloride from 0.1 mg mL^−1^ to 0.4 mg mL^−1^ and potassium sorbate from 0.02 mg mL^−1^ to 0.08 mg mL^−1^. Linear regression was used to process the calibration data. The correlation coefficients of linearity were 0.9995 for sotalol hydrochloride and 0.9995 for potassium sorbate, which indicate good correlation between the peak areas and the range of concentrations studied.

#### 3.3.4. Accuracy

The solutions for injection were prepared using a placebo and stock solution of standards instead of the oral preparation. Six solutions were prepared from each of the five preparations. Each solution was injected onto the column three times. Accuracy is reported as a parameter recovery with relative standard deviations.

#### 3.3.5. Selectivity

**Figure 2:**
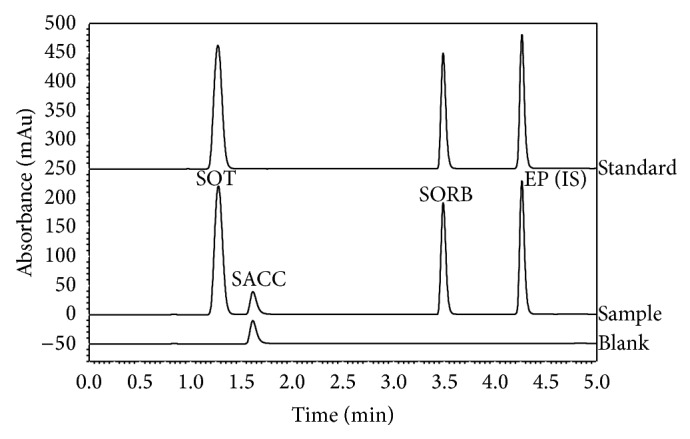
Chromatograms of the standard solution (SOT 0.2 mg mL^−1^, SORB 0.04 mg mL^−1^, and EP 0.08 mg mL^−1^), sample solution (1.000 mL of pharmaceutical preparation and 1.000 mL of stock solution of internal standard EP diluted to 25.00 mL), and blank solution (1.000 mL of placebo diluted to 25.00 mL); injection volume 5 *μ*L; mobile phase flow 1.3 mL min^−1^; linear gradient (ACN: 10% to 60% in 4 minutes); UV/Vis detector wavelength 237 nm; column oven 25°C.

The selectivity was determined by comparing the chromatograms of sample solutions, standard solution, and blank solutions.  Figure 2 shows that sotalol hydrochloride (i.e., the active substance), potassium sorbate (i.e., antimicrobial agent), and ethylparaben (i.e., internal standard) are all completely separated from each other and from the saccharine peak both in the standard solution and in the sample solution. No interference was observed.

#### 3.3.6. Robustness

Various buffer pH values and compositions of the mobile phase were tested. A mobile phase buffer with a pH ranging from 2.3 to 2.7 was used without remarkable changes in the accuracy (98.99–100.37%). A sodium dihydrogen phosphate dihydrate (pH 2.5; 17.7 mM)-ACN initial gradient ratio ranging from 92 : 8 to 89 : 11 (v/v) was used without remarkable changes in the accuracy (97.42–100.70%). However, the 88 : 12 ratio led to higher fluctuations in the retention times, and, therefore, this ratio is not recommended. All of the tested ratios ensured complete separation to the baseline for all of these compounds. The stability of the standard solution was tested at room temperature without light protection and at 5 ± 3°C light protected 24, 48, and 72 hours after its preparation. The accuracy of the peak areas for both storage conditions during the entire 72 hours was 99.32–100.62%.

## 4. Conclusions

The optimal chromatographic conditions for separation of an active substance sotalol hydrochloride, potassium sorbate, and other substances were achieved on an Ascentis Express C18 (100 × 4.6 mm, particles 2.7 *μ*m) solid core particles column and with a linear gradient elution at a flow rate of 1.3 mL min^−1^, using pH 2.5 phosphate buffer-ACN mixture (ACN~10–60%) as mobile phase and detection set to a wavelength of 237 nm. The method is rapid with a total analysis time of 4.5 minutes (+2.5 minutes of reequilibration). The sample preparation is a simple “dilute and shoot” method using an internal standard (ethylparaben). All measured parameters of the validation demonstrate the suitability of this new HPLC method for the analysis of oral liquid pharmaceutical preparations containing the above substances. The method was successfully employed in a stability evaluation of the four developed formulations with different composition, which are now already being used in the therapy of arrhythmias in pediatric patients. The method is also suitable for general quality control, that is, not only just for extemporaneous preparations containing the mentioned substances.

## Supplementary Material

Supplementary Material is providing summary of pharmaceutical products containing sotalol registered in EU and selected non-EU countries according to medicinal products databases of respective national competent authorities for human medicines.

## Figures and Tables

**Table 1:** Linear gradient.

*T* [min]	% *A* (buffer)	% *B* (ACN)
0.00	90	10
4.00	40	60
4.49	40	60
4.50	90	10
7.00	90	10

**Table 2:** Stock, IS, standard, sample, and blank solutions preparation.

Composition and process	Stock solution of standards	Stock solution of IS	Standard solution	Sample solution	Blank solution
Sotalol hydrochloride	~100.00 mg	—	—	—	—
Potassium sorbate	~20.00 mg	—	—	—	—
Ethylparaben	—	~100.00 mg	—	—	—
Stock solution of standards	—	—	1.000 mL	—	—
Stock solution of IS	—	—	1.000 mL	1.000 mL	—
Oral preparation (SOT 5 mg mL^−1^)	—	—	—	1.000 mL	—
Placebo of preparation (neither SOT nor SORB)	—	—	—	—	1.000 mL
Dissolvent ACN : water	50 : 50 (v/v)	50 : 50 (v/v)	30 : 70 (v/v)	30 : 70 (v/v)	30 : 70 (v/v)
Total volume	20.00 mL	50.00 mL	25.00 mL	25.00 mL	25.00 mL
Membrane filtration 0.22 *µ*m	—	—	Yes	Yes	Yes
Injection to the column	—	—	Yes (5 *µ*L)	Yes (5 *µ*L)	Yes (5 *µ*L)

**Table 3:** Validation parameters of formulations F1–F5.

	F1		F2		F3		F4		F5		Criterion
	SOT	SORB	SOT	SORB	SOT	SORB	SOT	SORB	SOT	SORB	
Repeatability *t*_*R*_ (% R.S.D.)^a^	0.00	0.00	0.00	0.00	0.00	0.00	0.00	0.00	0.00	0.00	*X* < 1%
Repeatability area (% R.S.D.)^a^	0.22	0.57	0.22	0.57	0.22	0.57	0.22	0.57	0.22	0.57	*X* < 1%
Theoretical plates per meter	11,810	282,650	11,810	282,650	11,810	282,650	11,810	282,650	11,810	282,650	—
Resolution^a^	—	18.39	—	18.39	—	18.39	—	18.39	—	18.39	*R*_*ij*_ > 1.5
Tailing factor^a^	1.10	1.23	1.10	1.23	1.10	1.23	1.10	1.23	1.10	1.23	*T* = 0.8–1.5
Precision (% R.S.D.)^b^	0.43	—	0.16	—	0.49	0.98	0.26	1.51	0.21	1.11	*X* < 5%
Linearity (correlation coefficient)^c^	0.9995	0.9995	0.9995	0.9995	0.9995	0.9995	0.9995	0.9995	0.9995	0.9995	*R* > 0.9990
Accuracy recovery (%)^b^	101.09	—	99.57	—	99.59	98.26	99.93	98.70	99.35	98.57	*X* = 100 ± 5%
Accuracy (% R.S.D.)^b^	0.58	—	0.85	—	1.44	2.27	0.71	2.07	0.57	1.14	*X* < 5%
Selectivity	No interference		No interference		No interference		No interference		No interference		No interference

^a^Six injections.

^b^Six samples, three injections of each sample.

^c^At 50, 75, 100, 134, 166, and 200% concentration levels.

SOT: sotalol hydrochloride.

SORB: potassium sorbate.

Fx: formulations with various excipients.

% R.S.D.: relative standard deviation in %.
