# Supplementary material for: Development of a Gradient HPLC Method for the Simultaneous Determination of Sotalol and Sorbate in Oral Liquid Preparations Using Solid Core Stationary Phase
Source: J Anal Methods Chem. 2015 Mar 24;2015:806736. doi: 10.1155/2015/806736 (PMC4388021; doi:10.1155/2015/806736)
Supplement: Supplementary file 1 — Supplementary Material is providing summary of pharmaceutical products containing sotalol registered in EU and selected non-EU countries according to medicinal products databases of respective national competent authorities for human medicines. [file 806736.f1.pdf]

Supplement. Pharmaceutical products containing sotalol registered in EU and selected non-EU countries [3]

	40 mg tablets	80 mg tablets	120 mg tablets	160 mg tablets	200 mg tablets	240 mg tablets	Solution for injection 10 mg/mL	Solution for injection 15 mg/mL	Oral liquid solution
EU									
Austria		●		●					
Belgium		●		●					
Bulgaria		●		●					
Croatia		●							
Cyprus		●							
Czech Republic		●		●					
Denmark	●	●		●					
Estonia		●							
Finland		●		●					
France		●		●					
Germany	●	●		●			●		
Greece		●		●					
Hungary		●		●					
Ireland		●		●					
Italy		●		●					
Latvia		●		●					
Lithuania									
Luxembourg				●					
Malta		●							
Netherlands	●	●		●					
Poland	●	●		●			●		
Portugal		●		●			●		
Romania		●		●					
Slovak Republic									
Slovenia		●							
Spain		●							
Sweden	●	●		●					
UK	●	●		●	●				
Non-EU									
Australia		●		●			●		
Canada		●		●					
USA		●	●	●		●		●	
